# Characterization of a Novel Endoplasmic Reticulum Protein Involved in Tubercidin Resistance in *Leishmania major*

**DOI:** 10.1371/journal.pntd.0004972

**Published:** 2016-09-08

**Authors:** Juliana Ide Aoki, Adriano Cappellazzo Coelho, Sandra Marcia Muxel, Ricardo Andrade Zampieri, Eduardo Milton Ramos Sanchez, Audun Helge Nerland, Lucile Maria Floeter-Winter, Paulo Cesar Cotrim

**Affiliations:** 1 Departamento de Fisiologia, Instituto de Biociências, Universidade de São Paulo, São Paulo, Brazil; 2 Departamento de Parasitologia, Instituto de Ciências Biomédicas, Universidade de São Paulo, São Paulo, Brazil; 3 Departamento de Biologia Animal, Instituto de Biologia, Universidade Estadual de Campinas, Campinas, Brazil; 4 Instituto de Medicina Tropical de São Paulo, Universidade de São Paulo, São Paulo, Brazil; 5 Department of Clinical Science, University of Bergen, Bergen, Norway; 6 Instituto de Medicina Tropical, Departamento de Moléstias Infecciosas e Parasitárias, Faculdade de Medicina, Universidade de São Paulo, São Paulo, Brazil; Bernhard Nocht Institute for Tropical Medicine, GERMANY

## Abstract

**Background:**

Tubercidin (TUB) is a toxic adenosine analog with potential antiparasitic activity against *Leishmania*, with mechanism of action and resistance that are not completely understood. For understanding the mechanisms of action and identifying the potential metabolic pathways affected by this drug, we employed in this study an overexpression/selection approach using TUB for the identification of potential targets, as well as, drug resistance genes in *L*. *major*. Although, TUB is toxic to the mammalian host, these findings can provide evidences for a rational drug design based on purine pathway against leishmaniasis.

**Methodology/Principal findings:**

After transfection of a cosmid genomic library into *L*. *major* Friedlin (LmjF) parasites and application of the overexpression/selection method, we identified two cosmids (cosTUB1 and cosTU2) containing two different *loci* capable of conferring significant levels of TUB resistance. In the cosTUB1 contained a gene encoding NUPM1-like protein, which has been previously described as associated with TUB resistance in *L*. *amazonensis*. In the cosTUB2 we identified and characterized a gene encoding a 63 kDa protein that we denoted as tubercidin-resistance protein (TRP). Functional analysis revealed that the transfectants were less susceptible to TUB than LmjF parasites or those transfected with the control vector. In addition, the *trp* mRNA and protein levels in cosTUB2 transfectants were higher than LmjF. TRP immunolocalization revealed that it was co-localized to the endoplasmic reticulum (ER), a cellular compartment with many functions. *In silico* predictions indicated that TRP contains only a hypothetical transmembrane domain. Thus, it is likely that TRP is a lumen protein involved in multidrug efflux transport that may be involved in the purine metabolic pathway.

**Conclusions/Significance:**

This study demonstrated for the first time that TRP is associated with TUB resistance in *Leishmania*. The next challenge is to determine how TRP mediates TUB resistance and whether purine metabolism is affected by this protein in the parasite. Finally, these findings may be helpful for the development of alternative anti-leishmanial drugs that target purine pathway.

## Author Summary

The identification of genes associated with drug resistance has contributed for understanding of the mechanisms of action of compounds against *Leishmania*, as well as, in the identification of the resistance mechanisms mediated by the proteins encoded by these genes. Differently from the mammalian host, *Leishmania* is unable to synthetize purine nucleotides *de novo* and must rescue preformed purines from its host. Due to this metabolic difference between host and parasite, the purine metabolism can be considered as a potential target for drug targeting. TUB is a toxic adenosine analog that was already demonstrated as effective against *Leishmania*. Using a strategy of gene overexpression after cosmid genomic library transfection, we isolated, mapped, sequenced and identified two genes involved in TUB resistance in *L*. *major*. In one of the cosmids, we identified NUPM1-like protein, an atypical multidrug resistance protein previously described in *L*. *amazonensis* involved in TUB resistance. The other cosmid contains a novel resistance marker involved in TUB resistance, described here as the TRP. Co-localization of TRP in the ER of LmjF and *in silico* structural predictions indicated that TRP might be an ER lumen protein. Our findings may be useful to elucidate the purine pathway in the parasite and to understand the role of TRP in the mechanism of TUB resistance.

## Introduction

*Leishmania* spp. are the causative agents of leishmaniasis, a parasitic protozoan disease that affects 12 million people worldwide with an estimated annual incidence of approximately 1 million, including both visceral and cutaneous cases [1].

The leishmaniasis chemotherapy is complicated because most of drugs used are expensive, toxic, and require long periods of supervised therapy [2]. Pentavalent antimonial is the WHO-recommended drug for the treatment of leishmaniasis; however, it has several side effects and reports of parasite resistance have been described worldwide [3]. Cases that are unresponsive to antimonial treatment are usually treated with amphotericin or pentamidine, although these drugs also have several side effects [3]. Miltefosine is the first effective oral drug developed to treat visceral leishmaniasis. It has been used in India for more than a decade [4] and an increase in the failure rate has been reported [5, 6].

Considering the limitations of the currently used chemotherapy and the lack of effective vaccines for the leishmaniasis, the identification of new drugs and vaccine approaches for the treatment of leishmaniasis is required. A rational strategy for chemotherapeutic exploitation in parasitic diseases can be developed, based on the identification of fundamental metabolic differences between parasite and host. New potential drug targets have been identified in molecular and biochemical studies to identify potential targets of the parasite that can be used in future therapies [7, 8].

An interesting pathway for exploration in the parasite is the purine metabolism. Purine nucleotides and their derivatives are precursors of a variety of cellular and metabolic processes, including energy production, cell signaling, synthesis of nucleic acids, modulation of enzymatic activities and synthesis of co-enzymes [9–11]. *Leishmania* and other protozoan parasites are unable to synthesize purine nucleotides *de novo* and must salvage them from the host [10]. This unique characteristic may be the basis for the susceptibility of *Leishmania* to purine analogs [12, 13]. Purine uptake in *Leishmania* is required for parasite viability during all life cycle stages [14]. Parasite nucleoside transporters, located on the plasma membrane, perform an essential function in uptake of purine nucleosides from the host into the parasite, which is the first step in the salvage process [15].

TUB is a toxic adenosine analog that is incorporated into nucleic acids in microorganisms and in mammalian cells. TUB has been previously described as a potential antiparasitic agent due to its inhibition of purine uptake in *Schistosoma mansoni*, *S*. *japonicum* [16, 17], *Trypanosoma gambiensi* [18] and *Leishmania* spp. [19, 20].

Considering the potential antiparasitic activity of TUB, in this study we aimed to identify the potential *loci* involved in TUB resistance in *L*. *major*. This knowledge is essential to understand the mechanism of action and resistance of this compound, as well as for identifying potential drug targets in the parasite. Accordingly, an overexpression/selection method with cosmid genomic libraries of LmjF was used to isolate two *loci* involved in TUB resistance [21]. One of the isolated cosmids (cosTUB1) contains a *locus* encoding NUPM1, a putative transcription-factor-like protein, previously described as toxic nucleoside resistance (TOR) [22]. TOR was described in *L*. *amazonensis* promastigote mutants resistant to TUB after drug selection *in vitro*, as an atypical multidrug resistance protein [22–24]. The other isolated cosmid (cosTUB2) contains a *locus* involved in TUB resistance following overexpression. This *locus* is not related to the *tor* gene or to other previously described *locus* involved in drug resistance in *Leishmania* [21]. Interestingly, these two *loci* are associated with two different resistance profiles: while *tor* also confers resistance to both inosine dialdehyde and allopurinol, the other *locus* confers resistance to inosine dialdehyde and hypersensitivity to allopurinol [21].

Based on these previous findings, in this study, we mapped, sequenced the genomic regions of two cosmids (cosTUB1 and cosTUB2) and identified the two genes related to TUB resistance in LmjF. TUB resistance gene in cosTUB1 encodes the previously described TOR protein, while the *locus* in cosTUB2 encodes a hypothetical protein. Of the 8,272 protein-coding genes predicted and annotated in LmjF genome, approximately 50% are annotated as hypothetical proteins; most of them are likely involved in essential cellular processes [25, 26]. Thus, the identification and characterization of TRP, may contribute for increasing the understanding of the purine pathway in *Leishmania* and the role of this protein in drug resistance mechanisms.

## Methods

### Parasite strain, culture and drugs

*Leishmania major* Friedlin (LmjF) strain (MHOM/IL/1980/Friedlin) promastigotes were grown at 25°C in M199 medium supplemented with L-glutamine, 10% heat-inactivated fetal calf serum, 0.25% hemin, 12 mM NaHCO_3_, 100 μM adenine, 40 mM HEPES, 50 U/mL penicillin and 50 μg/mL streptomycin. TUB, allopurinol, pentamidine, hygromycin B (HYG) and G418 were obtained from Sigma-Aldrich (St. Louis, MO, USA). Transfected parasites were cultured in M199 medium supplemented with increasing concentrations of HYG (125 to 500 μg/mL) or G418 (32 to 500 μg/mL), depending on the drug resistance marker.

### Cosmid libraries and transfections

Cosmids cosTUB1 and cosTUB2 associated with TUB resistance were previously isolated by an overexpression/selection strategy in LmjF as described by Cotrim et al. [21]. Briefly, two genomic libraries containing 30–40 kb inserts of genomic DNA from LmjF strain constructed in the shuttle vector cLHYG [27] were prepared by shearing or *Sau*3A partial digestion [21]. After transfection of these two cosmid libraries, parasites were plated on semisolid media in the presence of two independent concentrations of TUB (0.9 and 1.8 μM). A total of 39 colonies obtained after 10–15 days of incubation were then transferred to M199 liquid medium containing increasing concentrations of HYG (from 125 to 500 μg/mL) to increase the cosmid copy number [21]. Cosmid DNA was recovered from these primary TUB-resistant transfectants, used to transform into *Escherichia coli* DH5α strain and analyzed by restriction enzyme digestion [21]. Southern blot analysis confirmed the presence of two independent *loci* involved in TUB resistance, one containing *tor* gene and the other corresponding to a new *locus*.

To confirm the role in TUB resistance, cosTUB1 and cosTUB2 were transfected back into LmjF and after increasing cosmid copy number, tested for TUB resistance. Deletions of cosTUB1 and cosTUB2 were generated by partial digestion with *Kpn*I and *Apa*I, respectively, followed by self-ligation, to identify the gene(s) involved in TUB resistance. A pTRP construct was generated by total digestion of cosTUB2 with *Cla*I and *Eco*RV restriction enzymes, followed by ligation of the 3 kb fragment into the pSNBR vector [28], previously digested with the same enzymes. Transfections were performed as described by Coburn et al. [29]. Promastigotes from the late logarithmic phase were harvested, washed and resuspended in electroporation buffer. A total of 20–40 μg of cosmid and plasmid DNA was mixed on ice with 4x10^7^ cells in a 2-mm cuvette and subjected to electroporation (500 μF, 2.25 kV/cm) using a Bio-Rad Gene Pulser apparatus. Mock transfection was performed in absence of cosmid or plasmid DNA for the negative control while the transfection with cosmid or plasmid DNA of empty vector was performed as control. Transfected parasites were kept on ice for 10 minutes and then transferred to 10 mL M199 medium. The antibiotics HYG or G418 were added after 24 hours, depending on the drug resistance marker.

### Analysis of drug susceptibility

For drug susceptibility analysis, promastigotes (10^6^ promastigotes/mL) were incubated at 25°C in the presence of increasing TUB concentrations for 72 hours, and then the number of parasites was determined using a Coulter T890 (Beckman, CA, USA). The 50% inhibitory concentration (IC_50_) was measured at the time when control cultures lacking the drug had reached the late logarithmic phase of growth [30, 31]. The results are expressed as the means ± standard error. Statistical analysis was performed using the non-parametric Kruskal-Wallis test and p < 0.05 was considered significant.

### Identification of TUB-resistance-related *loci* and nucleotide sequencing

To identify the genomic region of interest in *L*. *major* genome databases, a 1.0 kb *Eco*RI fragment from cosTUB1 and a 1.0 kb *Eco*RV fragment from cosTUB2 were subcloned into a pUC-π vector. The subcloned fragments were sequenced using a MegaBACE 1000 automated sequencer (GE Healthcare, UK) with DYEnamic Dye Terminator kit (GE Healthcare, UK), according to the manufacturer’s instructions. Analyses of the nucleotide sequences were performed using Lasergene Software (DNASTAR, Inc.) and Clone Manager 9 Software. Sequence data for the remaining regions were obtained from LmjF GeneDB [25]. Nucleotide sequences obtained were used to map the genomic region corresponding to the two different *loci* using LmjF database. *In silico* analyses were also conducted to estimate the insert sizes of both cosmids after digestion with different restriction enzymes.

### *In silico* analysis and protein prediction

*In silico* analysis of the genomic regions involved in TUB resistance was performed using DNASTAR and Clone Manager 9 Software. BLAST searches of LmjF GeneDB [25] and TriTrypDB [32] were performed using the standard settings. Multiple alignments were performed using the Constraint-based Multiple Protein Alignment Tool (Cobalt) [33]. Prediction of transmembrane domains was performed with the TMHMM Server [34, 35] while the predictions of protein function, sub-cellular localization and the tridimensional TRP structure were performed using ProtFun [36, 37], TargetP Server [38, 39] and Phyre^2^ [40], respectively.

The sequence data described in this paper are available under the following accession numbers: XM_001685128.1 for gene ID LmjF.31.1940 (*nupm1*), and XM_001685135.1 for gene ID LmjF.31.2010 (*trp*). All sequence data are also available at www.tritrypdb.org.

### Total RNA isolation and RT-qPCR

Total RNA from promastigotes in stationary growth phase and during the growth curve on days 3, 5, 7 and 9, were isolated using TRIzol reagent (Life Technologies, Carlsbad, CA, USA), according to the manufacturer’s instructions. RNA samples were treated with DNase I (Thermo Scientific, Lithuania, EU) and RNA concentration and purity were determined using a spectrophotometer at A260/A280 (Nanodrop ND1000, Thermo Scientific, USA). Reverse transcription was performed using 2 μg of total RNA as template, reverse transcriptase and random primers (cDNA synthesis kit, Thermo-Scientific, Canada), according to the manufacturer’s instructions. Equal amounts of cDNA were run in triplicate in a total volume of 25 μL containing Power SYBR Green Master Mix (Life Technologies, Warrington, UK) and the following primers (10 μM): TRP_F 5´-CGGTGTAGATGAACCAGCAGTAG-3´, TRP_R 5´-CTCACAGAGGGATTTCGAGAGTG-3´, GAPDH_F 5´-AACGAGAAGTTCGGCATAGTCGAG-3´ and GAPDH_R: 5´-ACTATCCACCGTCTTCTGCTTTGC-3´. The mixture was incubated at 94°C for 5 minutes, followed by 40 cycles at 94°C for 30 sec, 64°C for 30 sec and 72°C for 30 sec. A negative control in the absence of reverse transcriptase was included in RT-qPCR assays for check DNA contamination in RNA samples. Reactions were carried out using an Exicycler 96 (Bioneer, Daejeon, Korea). The copy number of the target gene (*trp*) and housekeeping gene (*gapdh*) were quantified in three biological replicate samples, considering the molar mass concentration, according to a standard curve generated from a ten-fold serial dilution of a quantified and linearized plasmid containing the target fragment for each quantification test. The normalized *trp/gapdh* ratio of the absolute number of molecules of each target was used as parameter of the relative expression of *trp* in the cosTUB2 and pTRP transfectants relative to LmjF or the line transfected with the empty vector (pSNBR or cLHYG). Analyses were performed using Analysis Exicycler3 Software (Bioneer, Daejeon, Korea).

### Cloning, expression and purification of TRP, and production of a rabbit anti-TRP polyclonal antibody

The open reading frame (ORF) of gene *trp* (*LmjF*.*31*.*2010)* was amplified by PCR using the following primers containing the restriction enzyme sites for *Bam*HI and *Not*I (underlined): TRP_F_*Bam*HI 5´-GGATCCATGGAGTGCATCAACCAAGAGAGC-3´ and TRP__R_*Not*I 5´-GCGGCCGCTCACATGGCACAGATAAACACC-3´. The amplified fragment was cloned into the pET28a (Novagen, USA) expression vector and sequenced to confirm the insertion direction. The pET-TRP plasmid obtained was then used to transform into *E*. *coli* (BL21(DE3)CodonPlus-RIL). Selected clones were grown aerobically at 37°C in LB medium containing kanamycin (30 μg/mL) and chloramphenicol (35 μg/mL) to a culture OD_600_ 0.6–0.8. pET-TRP expression was induced by 1 mM of isopropyl-β-D-thiogalactopyranoside. After induction, the culture was lysed by sonication (Sonics–VCX500) with 20 mM sodium phosphate, 500 mM sodium chloride and 5 mM imidazole. Lysed samples were clarified by centrifugation at 10,000 x g, for 15 minutes at 4°C, and inclusion bodies were solubilized with 20 mM sodium phosphate, 500 mM sodium chloride, 5 mM imidazole and 8 M urea. Recombinant TRP was obtained by affinity chromatography with a 1 mL HisTrap HP column (GE Healthcare, Uppsala, Sweden). The purified recombinant TRP was analyzed by SDS-PAGE and then used to produce a rabbit polyclonal anti-TRP antibody by Proteimax Biotechnology (Sao Paulo, Brazil).

### Western blot analysis

Approximately 10^7^ promastigotes in the stationary growth phase and during the growth curve on days 3, 5, 7 and 9 were washed with PBS and then lysed with lysis buffer (100 mM Tris-HCl pH 7.5, 2% Nonidet P40, 1 mM PMSF and protease inhibitor cocktail (Sigma-Aldrich, St Louis, MO, USA)). Cells were disrupted by ten freeze/thaw cycles in liquid nitrogen and 42°C, and were then cleared of cellular debris by centrifugation at 12,000 x g for 15 minutes at 4°C. Equal amounts of total protein (25 μg) were solved using SDS-PAGE and transferred to a nitrocellulose membrane (Hybond-C, Amersham Biosciences, Buckinghamshire, England) using a Trans-Blot SD apparatus (Bio-Rad, USA). The membrane was incubated with Blocking Buffer (LI-COR Bioscience, Lincoln, NE, USA) and then with anti-TRP serum (1:2000 dilution), overnight, at 4°C. After incubation with primary antibody, the membrane was incubated with biotin anti-rabbit antibody (Santa Cruz Biotechnology, CA, USA) (1:1000 dilution) for 1 hour at room temperature and then with streptavidin (Santa Cruz Biotechnology, CA, USA) (1:2000 dilution) for 30 minutes at room temperature for biotin-streptavidin binding. Anti-α-tubulin (Sigma-Aldrich, St. Louis, MO, USA) (1:1000 dilution) was used to normalize the amount of protein in the blot. All steps were followed by washing 3 times with PBS. The membranes were scanned using an Odyssey CLx apparatus (Li-COR, Lincoln, NE, USA) in both 700 and 800 nm channels using an Odyssey System. Odyssey Imaging CLx instrument was used at the 5/5 intensity setting (700/800 nm). Quantification of the protein level was performed with Image Studio2.1 Software (Li-COR, Lincoln, NE, USA). TRP target band densities were normalized against α-tubulin for blotting comparisons in LmjF, cosTUB2 and pTRP transfectants. Statistical analysis was conducted using the Mann-Whitney U-test, and p < 0.05 was considered significant for three independent experiments.

### Confocal immunolocalization

Approximately 10^6^ promastigotes of LmjF and pTRP transfectants in the stationary growth phase were washed with PBS and adhered to cover slips treated with poly-L-lysine (Sigma-Aldrich, St. Louis, MO, USA) for 15 minutes. The cells were then fixed with 3% paraformaldehyde for 10 minutes and treated with 50 mM ammonium chloride for 10 minutes. The fixed cells were permeabilized and blocked with 0.1% Triton X-100 and 0.1% BSA in PBS for 10 minutes at room temperature. To analyze sub-cellular TRP localization, anti-TRP polyclonal antibody (1:100 dilution) was visualized using an anti-rabbit secondary antibody conjugated to Alexa488 (Life Technologies, Carlsbad, CA, USA) (1:500 dilution). Anti-BiP/GRP78 (BD Bioscience, Iowa, USA) (1:500 dilution) was visualized using an anti-mouse secondary antibody conjugated to Alexa594 (Life Technologies, Carlsbad, CA, USA) (1:500 dilution). Nuclear and kinetoplast DNA were labeled using DAPI. Each step was followed by washing with PBS 10 times. The coverslips were mounted in ProLong media (Life Technologies, Carlsbad, CA, USA). All imaging was performed at the Molecular Imaging Center (MIC) of the University of Bergen, using a Zeiss LSM 510 Meta confocal microscopy. Co-localization images were edited using Photoshop 6.

## Results

### Tubercidin resistance in cosTUB1 is due to the *tor* gene

Using the overexpression/selection strategy described by Cotrim et al. [21], a library of 17,900 independent genomic cosmid transfectants in LmjF were recovered from semisolid plates in two TUB concentrations. Thirty-nine colonies showing differential survival were recovered and then analyzed by restriction enzyme digestion. cosTUB1a and cosTUB1b were recovered each one from a single colony, while cosTUB2 was recovered from several colonies [21].

Southern blot analysis demonstrated that cosTUB1a and cosTUB1b were involved in TUB resistance conferred by the TOR protein [21]. This protein has been previously described as related to TUB resistance in selected *L*. *amazonensis* promastigote mutants [22, 24]. Since cosTUB1a and cosTUB1b were referred to the same TUB resistance gene, we decided to examine only cosTUB1a followed of mapping, functional and sequencing analysis.

Parasites transfected with the cosmid cosTUB1 showed moderate TUB resistance (1.95-fold resistance) compared with the LmjF (S1 Table). To map the gene likely involved in the resistance phenotype, a set of deletions was generated using the restriction enzyme *Kpn*I. Four independent deletions were generated, transfected back into LmjF and amplified by HYG selection. Transfected parasites with the deletions cosTUB1-Δ*Kpn*I-III and cosTUB1-Δ*Kpn*I-IV exhibited 2.04- and 2.82-fold resistance, respectively, compared with LmjF parasites (S1 Fig and S1 Table). No significant differences in resistance were observed among the other deletions compared with LmjF or with transfected parasites carrying the empty vector cLHYG (S1 Table).

To identify the gene of cosTUB1 involved in TUB resistance, a 1.0 kb *Eco*RI fragment from this cosmid (S1 Fig) was sub-cloned into the pUC-π vector and sequenced. *In silico* analysis revealed that the cosTUB1 insert corresponds to a genomic DNA region from chromosome 31 of LmjF containing five ORFs (S1 Fig). Two of which encode hypothetical proteins (LmjF.31.1910 and LmjF.31.1920), and the other three encode peptidase m20/m25/m40 family-like protein (LmjF.31.1890), dihydrouridine synthase (LmjF.31.1930) and transcription-factor-like NUPM1 protein (LmjF.31.1940) (S1 Fig).

According to the map and the functional analysis, the 3.0 kb fragment represented in the deletion cosTUB1-Δ*Kpn*I-IV, contained the *locus* likely involved in TUB resistance (S1 Fig). In this region, we identified the gene that encodes the transcription-factor-like NUPM1 protein (LmjF.31.1940) (accession number XP_001685180.1) (S1 Fig). This protein has 77% similarity to NUPM1 of *L*. *amazonensis*, which has been previously described as TOR protein and it is related to TUB resistance in selected *L*. *amazonensis* promastigote mutants [22, 24]. According to TriTrypDB, *nupm1* encodes a 53.1 kDa protein with only one predicted transmembrane domain and no other predicted domain (S2 Fig).

### Tubercidin resistance in cosTUB2 is due to the *trp* gene

**Fig 1:**
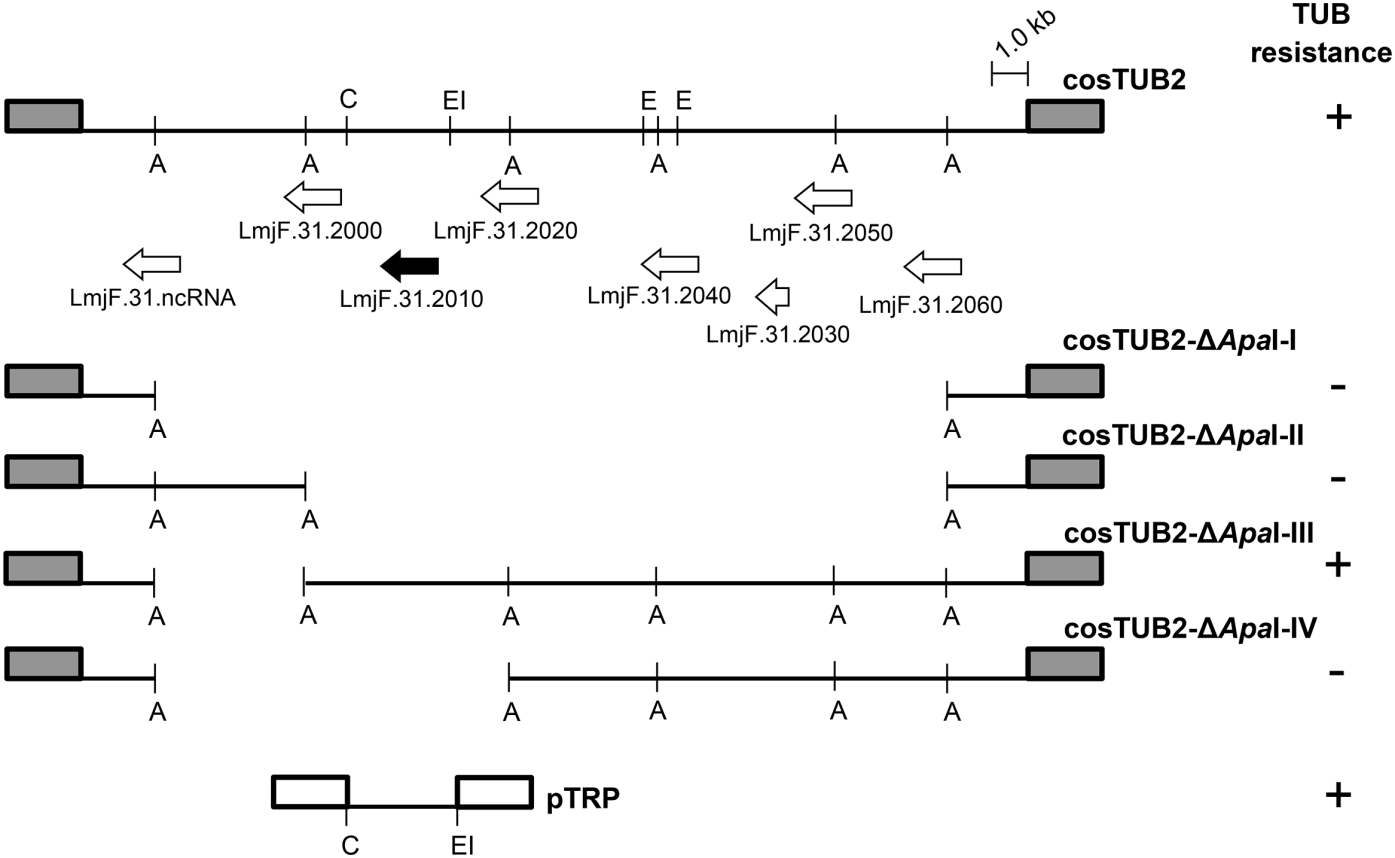
Restriction map of cosTUB2 and functional analysis. Linear representation of the cosTUB2 restriction map and the four deletions generated by partial digestion with *Apa*I (A). The restriction sites of *Cla*I (C), *Eco*RI (EI) and *Eco*RV (E) are also indicated in the Figure. TUB resistance is indicated by (+). The white arrows indicate the coding regions of the hypothetical proteins (LmjF.31.2040, LmjF.31.2050 and LmjF.31.2060), non-coding RNA (LmjF.31.ncRNA), glycoprotein-like (GP63-like) (LmjF.31.2000), succinyl-diaminopimelate-desuccinylase-like protein (SDD-like) (LmjF.31.2020) and ubiquitin-fusion protein (LmjF.31.2030). The black arrow indicates the *TRP* gene (LmjF.31.2010). The shaded boxes represent the cLHYG vector and the blank box represents the pSNBR vector.

The other cosmid, cosTUB2, was recovered 37 times and presented a different resistance profile compared with cosTUB1. It conferred a 3.78-fold increase in resistance compared with LmjF (Table 1). Using the same strategy as that described for cosTUB1, we mapped the likely gene involved in the resistance phenotype through the generation of a set of deletions with the restriction enzyme *Apa*I (Fig 1). Four independent deletions were generated, transfected back into LmjF and then selected using HYG selection. Parasites transfected with the cosTUB2-Δ*Apa*I-III deletion exhibited 2.0-fold resistance compared with LmjF (Fig 1 and Table 1). No significant difference was observed among the transfectants containing the other three deletions compared with LmjF or with transfected parasites carrying the empty vector cLHYG (Table 1).

**Table 1:** TUB resistance profiles of cosTUB2 and its respective deletions compared with LmjF parasites.

Cell line	IC_50_ ^(a)^ (μM)	fold ^(b)^ resistance	*p* value ^(c)^	TUB resistance
LmjF	0.23 ± 0.09	-	-	no
cLHYG	0.26 ± 0.06	1.13	ns	no
pSNBR	0.30 ± 0.03	1.30	ns	no
cosTUB2	0.87 ± 0.34	3.78	2x10^-6^	yes
cosTUB2-Δ*Apa*I-I	0.27 ± 0.05	1.17	ns	no
cosTUB2-Δ*Apa*I-II	0.21 ± 0.07	0.91	ns	no
cosTUB2-Δ*Apa*I-III	0.46 ± 0.05	2.00	2x10^-5^	yes
cosTUB2-Δ*Apa*I-IV	0.26 ± 0.09	1.13	ns	no
pTRP	0.44 ± 0.08	1.91	0.002	yes

^(a)^ Mean ± standard deviation of the IC_50_ values of at least three independent experiments for each indicated cell line.

^(b)^ Fold-resistance is the ratio of the IC_50_ value for the respective transfected line and LmjF.

^(c)^ *p* values versus LmjF according to the non-parametric Kruskal-Wallis test.

(ns)–not significant.

To map the genomic region corresponding to the *locus* involved in TUB resistance, a 1.0 kb *Eco*RV fragment from cosTUB2 (Fig 1) was sub-cloned into the pUC-π vector and sequenced. Sequence analysis indicated that this DNA fragment corresponded to a second region of chromosome 31 of *L*. *major*. *In silico* analysis indicated the presence of eight ORFs in the 30 kb genomic region of cosTUB2. Four of these genes were annotated as encoding hypothetical proteins (LmjF.31.2010, LmjF.31.2040, LmjF.31.2050 and LmjF.31.2060), while the other four genes encoded a non-coding RNA (LmjF.31.ncRNA), a glycoprotein-like (GP63-like) (LmjF.31.2000), a succinyl-diaminopimelate-desuccinylase-like (SDD-like) protein (LmjF.31.2020) and an ubiquitin-fusion protein (LmjF.31.2030) (Fig 1). *In silico* data associated with genomic mapping and functional analysis indicated that the gene *LmjF*.*31*.*2010*, located in the 9.6 kb fragment of cosTUB2 and also in cosTUB2-Δ*Apa*I-III (Fig 1), encodes a hypothetical protein that could be involved in TUB resistance.

To confirm *LmjF*.*31*.*2010* as the gene involved with TUB resistance, we sub-cloned the region encompassing the *LmjF*.*31*.*2010* gene (a 3 kb fragment from cosTUB2 digested with *Cla*I-*Eco*RI) into pSNBR vector, previously digested with the same enzymes (Fig 1). This construct (pTRP) was transfected back into LmjF and the amplification and overexpression of this gene was obtained by increasing the concentration of G418. As expected, the pTRP transfectants exhibited 1.91-fold resistance compared with LmjF (Table 1), confirming the role of *LmjF*.*31*.*2010* gene in TUB resistance.

### *trp* mRNA expression

**Fig 2:**
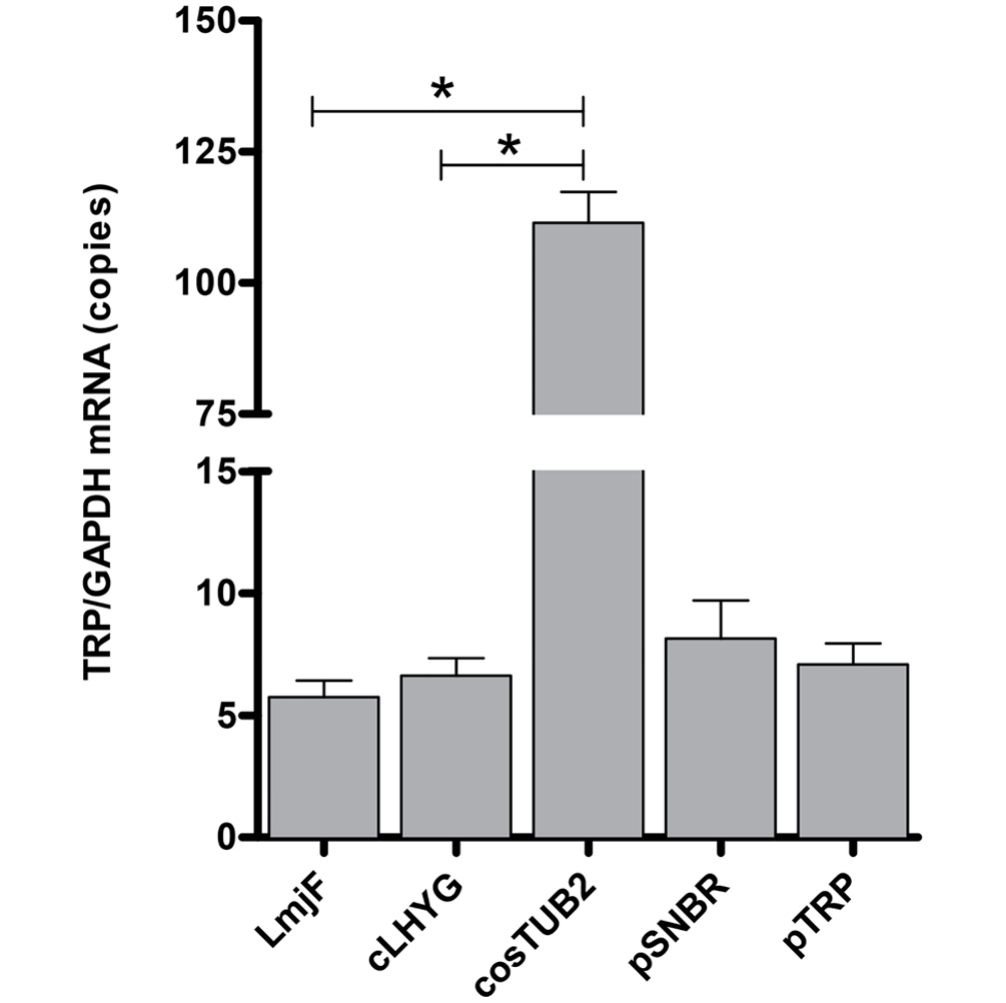
Evaluation of *trp* mRNA expression. *trp* mRNA expression in promastigotes of LmjF, and the lines transfected with the cLHYG vector, cosTUB2, pSNBR vector and pTRP were determined by *trp*-specific RT-qPCR. Data were based on quantification of the target and were normalized by *gapdh* expression. (*) p < 0.0001, compared with the line transfected with the vector cLHYG or with LmjF. The values are the mean ± SEM of three independent biological replicates.

The *trp* mRNA expression in LmjF promastigotes was measured by RT-qPCR. Quantification of *trp* transcripts in the cosTUB2 and pTRP transfectants relative to LmjF or to the transfected line with the empty vector (cLHYG or pSNBR) was performed using *trp* gene as target. The data were normalized by the amount of *gapdh* transcript. Both sequences corresponded to single copy genes. As shown in Fig 2, the cosTUB2 transfectant exhibited 19.4- and 16.8-fold increase in *trp* mRNA expression compared with LmjF and with cLHYG transfectant, respectively. In contrast, no significant change in *trp* mRNA expression was observed between the pTRP transfectant and LmjF or pSNBR transfectant (Fig 2).

Additional data obtained during the time-course of growth curve of LmjF promastigotes demonstrated that *trp* transcript expression was increased in the day 3, in the logarithimic growth phase. As shown in S3 Fig, *trp* mRNA transcript expression was 2-fold increase on day 3 compared with days 5, 7 and 9. The same profile was observed at protein levels, with an increase of 1.5-fold on day 3 compared with days 5, 7 and 9 (S3 Fig).

### TRP expression in promastigotes

**Fig 3:**
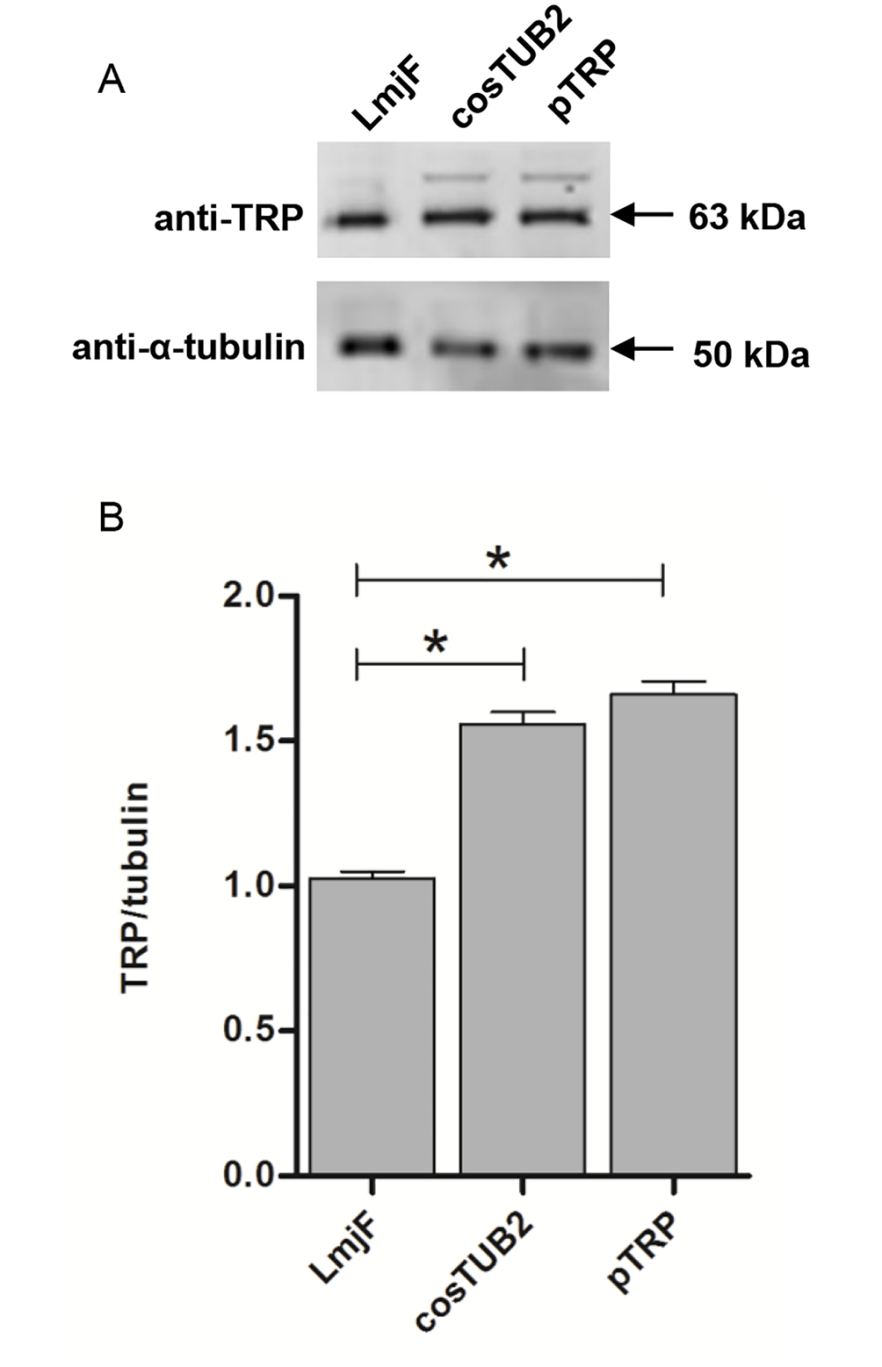
TRP expression in transfectant lines, as determined by Western blotting. Total extracts of promastigotes in the stationary phase for LmjF, cosTUB2 and pTRP transfectants were lysed and the proteins were separated by SDS-PAGE, transferred to nitrocellulose membrane and immunoblotted with an anti-TRP polyclonal antibody. An anti-α-tubulin antibody was used as a control. The images were scanned using an Odyssey CLx imaging system (Li-COR). (A) Western blot analysis of LmjF, cosTUB2 and pTRP transfectants. (B) The bands were quantified using Image Studio2.1 Software (Li-COR) and the results for TRP were normalized against α-tubulin for blotting comparisons. Statistical analysis was performed using Mann-Whitney U test. (*) p < 0.05, compared with LmjF.

Western blot analysis of cell lysates from LmjF and parasites transfected with cosTUB2 and pTRP were performed using an anti-TRP polyclonal antibody. As shown in Fig 3, the signal intensity of TRP expression was significantly increased in the cosTUB2 and pTRP lysates compared with LmjF lysate.

### Predictions for TRP

**Fig 4:**
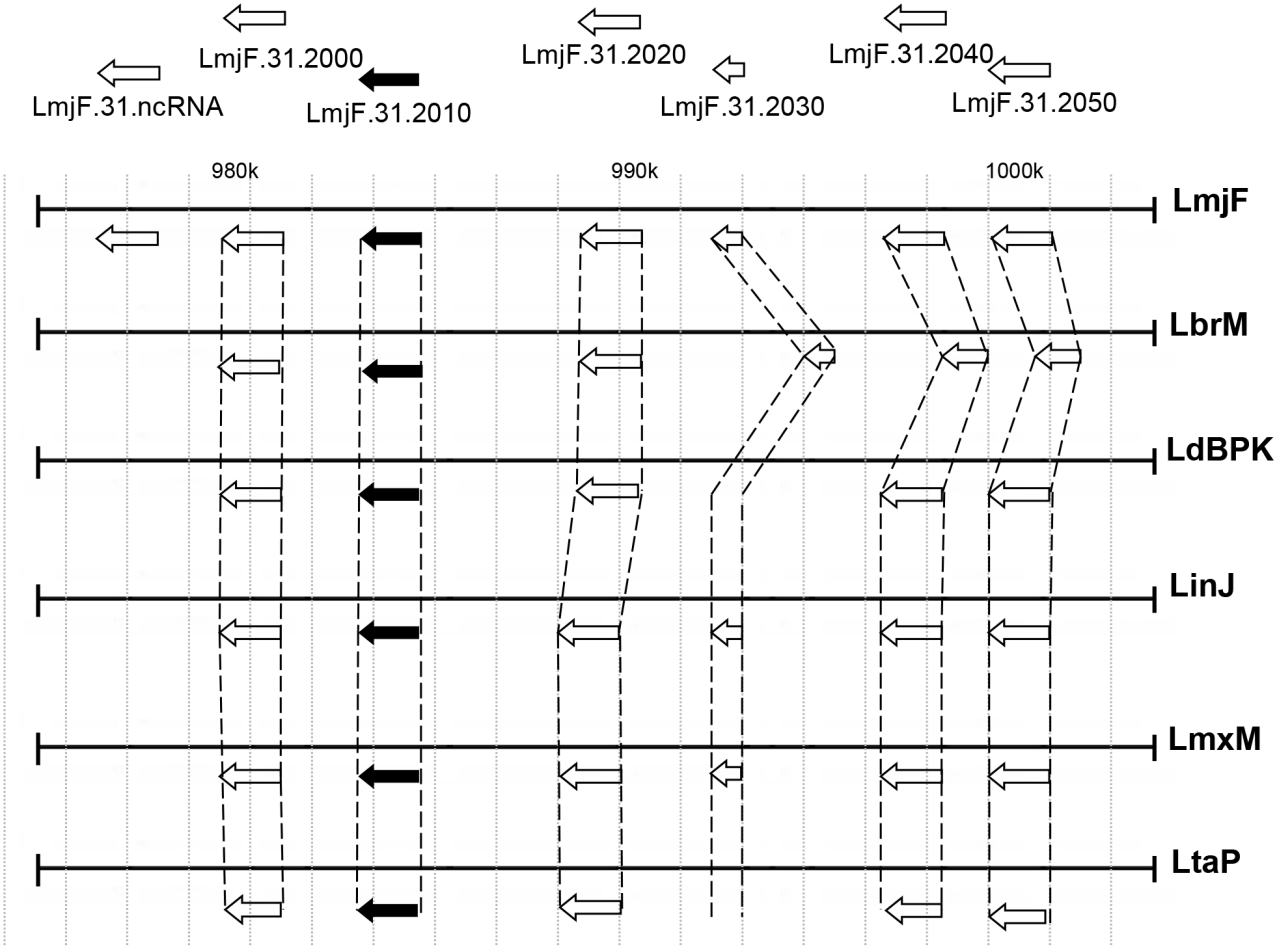
Mapping and alignment of the genomic region around *trp* gene for six *Leishmania* species. Map of the genomic region of cosTUB2 from *L*. *major* Friedlin (LmjF) and localization of the *trp* gene (LmjF.31.2010) in comparison with the following *Leishmania* spp., according to TriTrypDB: *L*. *(V*.*) braziliensis* MHOM/BR/75/M2904 (LbrM), *L*. *(L*.*) donovani* BPK282A1 (LdBPK), *L*. *(L*.*) infantum* JPCM5 (LinJ), *L*. *(L*.*) mexicana* MHOM/GT/2001/U1103 (LmxM), *L*. *(L*.*) tarentolae* Parrot-TarII (LtaP). The white arrows indicate the coding regions of hypothetical proteins (LmjF.31.2040, LmjF.31.2050 and LmjF.31.2060), non-coding RNA (LmjF.31.ncRNA), glycoprotein-like 63 (GP63-like) (LmjF.31.2000), succinyl-diaminopimelate-desuccinylase-like (SDD-like) protein (LmjF.31.2020) and the ubiquitin-fusion protein (LmjF.31.2030). The black arrow indicates the *trp* gene (LmjF.31.2010).

Sequence analysis of the *trp* gene (*LmjF*.*31*.*2010*) (accession number XP_001685187.1) indicated that it encodes a 63.4 kDa protein. It is conserved in the genus *Leishmania*, and it is not present in other trypanosomatids, such as *Trypanosoma brucei* and *T*. *cruzi* (Fig 4). This gene encodes for a protein that contains just one hypothetical transmembrane domain and no other putative conserved domain or peptide signal (S4 Fig). Interestingly, multiple sequence alignments revealed the presence of 11 amino acids specific to *L*. *braziliensis* (LbrM) (S4 Fig). Indeed, TRP of *L*. *major* contains approximately 85% of similarity with its orthologs of the subgenus *Leishmania* and 54% of similarity with its ortholog of *L*. *(Viannia) braziliensis* (S4 Fig).

Additional *in silico* data based on the Protein Functional Category and Enzyme Class Database (ProtFun Server), which predicts cellular role and enzyme class based on gene ontology, indicated that TRP is an enzyme involved in the purine and pyrimidine pathway. *In silico* analysis of TRP using the TargetP 1.1 Server, which predicts the sub-cellular localization of eukaryotic proteins revealed the absence of any sequence corresponding to a mitochondrial targeting peptide.

**Fig 5:**
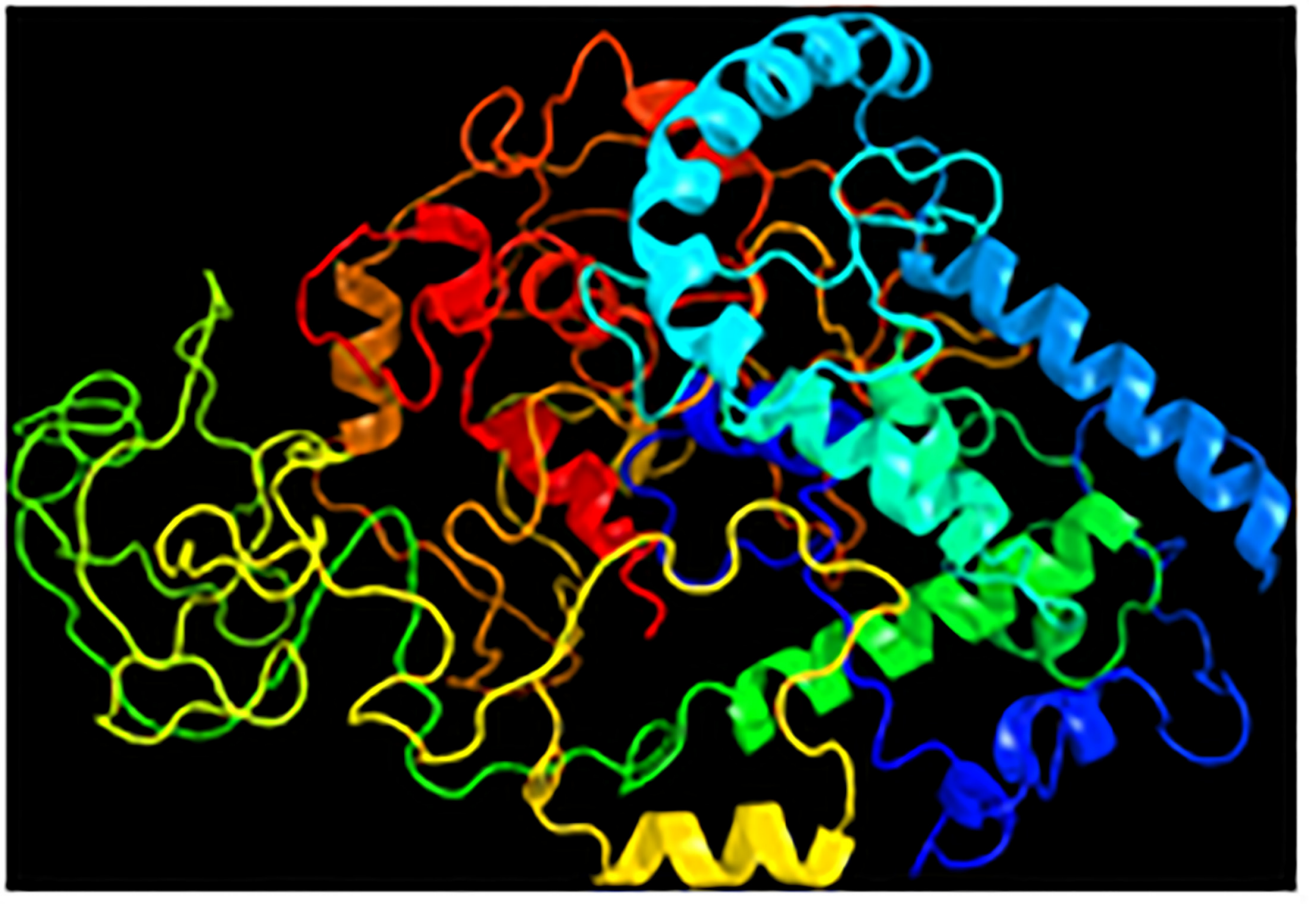
Structural prediction of the TRP. Protein modeling, prediction and analysis, according to Phyre2 web portal for TRP. The predicted protein contains 36% alpha helix, 6% beta strand and 3% transmembrane helix, in addition to multidrug efflux transporter, hydrolase/transport protein and transferase folds.

3D protein prediction was performed by submitting the amino acid sequence to the Phyre2 web portal for protein modeling, prediction and analysis. The predicted model of TRP based on heuristics to maximize confidence, percent identity and alignment coverage is shown in Fig 5. Some disordered regions were observed, but the prediction showed 90% confidence. Some regions have interesting folding patterns with similarities to transmembrane helices, multidrug efflux transporter, hydrolase/transport protein and transferase (Fig 5).

### Immunolocalization of TRP

**Fig 6:**
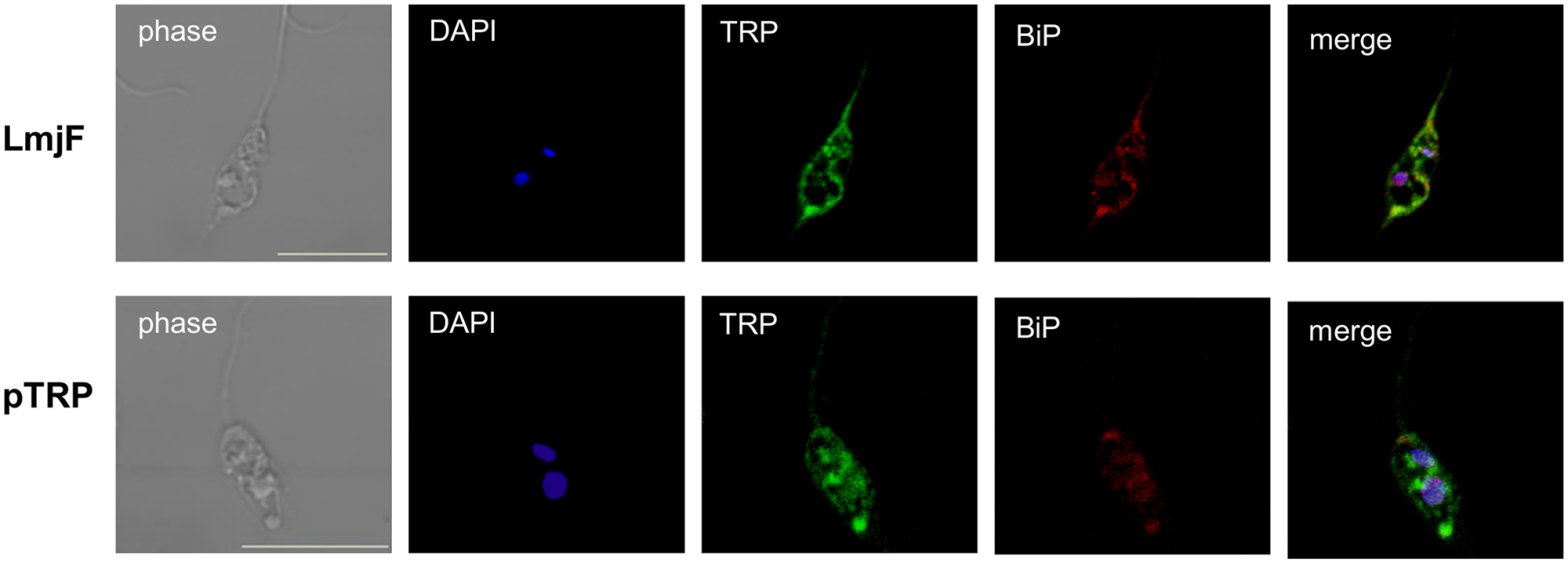
Cellular localization of TRP in *L*. *major*. Promastigotes in the stationary phase of LmjF and transfected line with pTRP showing phase-contrast image, DNA staining using DAPI (blue), anti-TRP polyclonal antibody visualized with an anti-rabbit secondary antibody conjugated to Alexa488 (green), anti-BiP/GRP78 visualized with an anti-mouse secondary antibody conjugated to Alexa594 (red) and merged image. All images were acquired using a Zeiss LSM confocal microscope. Bar 10μm.

For cellular immunolocalization of TRP, we used antibodies against the recombinant TRP. Considering our hypothesis that TRP is an ER protein, we used an ER marker (anti-BiP/GRP78). Confocal microscopy showed that TRP is co-localized in ER in stationary phase promastigotes of LmjF and of pTRP transfected line (Fig 6).

### Analysis of cross-resistance profiles against anti-leishmanial drugs

We next analyzed whether the transfected lines overexpressing the *trp* gene were resistant to other drugs. Interestingly, the cosTUB2 and pTRP transfectants showed cross-resistance to pentamidine, with 2.0- and 5.0-fold resistance, respectively, compared with LmjF (Table 2). No cross-resistance to allopurinol was observed. Indeed, the cosTUB2 transfectants were more sensitive to allopurinol.

**Table 2:** Cross-resistance profiles of cosTUB2 and pTRP transfectants compared with LmjF parasites.

		IC_50_ ^(a)^		fold-resistance ^(b)^	
Drug	LmjF	cosTUB2	pTRP	cosTUB2	pTRP
tubercidin (μM)	0.23 ± 0.09	0.87 ± 0.34	0.44 ± 0.08	3.78	2.00
allopurinol (μg/mL)	34.6 ± 6.05	19.0 ± 1.41	ND	0.54	-
pentamidine (μg/mL)	0.65 ± 0.07	1.35 ± 0.04	3.26 ± 0.60	2.03	5.01

^(a)^ Mean ± standard deviation of IC_50_ values from at least three independent experiments for each indicated line.

^(b)^ Fold-resistance is the ratio of the IC_50_ value for the transfectants and LmjF.

(ND)—Not done.

## Discussion

The molecular mechanism of action of compounds used in leishmaniasis treatment is not well known. Overexpression/selection methods have been used for identification of drug targets and potential drug resistant genes [21, 30, 31, 41]. Because of the limited knowledge about the purine metabolism in *Leishmania*, we proposed in this study to elucidate the purine pathway in *Leishmania* examining the resistance phenotype after transfection of a cosmid genomic library, followed by drug pressure using TUB. This drug has been demonstrated to have potent activity against promastigotes forms of *L*. *amazonensis*, *L*. *braziliensis*, *L*. *infantum chagasi* and *L*. *major* [19, 20]. The same antiparasitic efficacy has also been reported *in vitro* against intracellular amastigotes and *in vivo* infection against *L*. *amazonensis* when the drug was associated with the specific inhibitor of nucleoside transport for mammalian cells, the nitrobenzylthioinosine (NBMPR) [20].

Two cosmids conferring TUB resistance, cosTUB1 and cosTUB2, were isolated from transfected parasites with a genomic library constructed in the cLHYG vector [21]. The gene related to TUB resistance in cosTUB1 provided 2.82-fold resistance compared with LmjF. *In silico* analysis of the genomic region in *L*. *major* GeneDB revealed that cosTUB1 is located on chromosome 31 and that 1,455 base pair gene present in the 3.0 kb fragment encodes NUPM1-like protein. A previous study first referred to this protein as TOR because it was involved in TUB resistance in selected *L*. *amazonensis* mutants [23]. According to these authors, *L*. *amazonensis* becomes resistant to TUB by decreasing the capacity to accumulate this exogenous purine. Later, it was suggested that the decrease was related to the reduction in the activity of purine transporters [24]. The TOR protein mediates resistance by redirecting the adenosine permease from the plasma membrane to the multi-vesicular tubule lysosome [24] and TUB resistant parasites overexpressing *tor* are unable to uptake the toxic purine and become resistant to the drug [24]. Moreover, TOR could act at the protein level and affect the activity and/or the amount of transporters and other proteins [24]. The protein has similarities to Oct-6, a mammalian transcription factor of the Pou family [24, 42]. Members of the Oct family bind to the octamer motif, a *cis*-acting regulatory element enhancer and stimulate transcription via this octamer motif [42].

In contrast to TOR identification, we could not find any previously described gene in cosTUB2 related to TUB resistance. Similar to cosTUB1, the cosTUB2 insert contains a genomic region of chromosome 31, but the region is distinct from that one in cosTUB1. According to the restriction map and functional analysis, the 9.6 kb fragment present in both cosTUB2 and the cosTUB2-Δ*Apa*I-III deletion was suggested to be the region related to TUB resistance. *In silico* analysis showed that this region coded for a hypothetical protein (LmjF.31.2010), a succinyl-diaminopimelate-desuccinylase-like (SDD-like gene) and part of the glycoprotein 63 (GP63-like). GP63-like is the major surface glycoprotein in *Leishmania* promastigotes, beside lipophosphoglycan (LPG) and GIPLs [43]. Several functions of GP63 have been described in the vertebrate host, including cell adhesion to mammalian cells. It is predominantly expressed in the form present in the insect host and unknown relationship with drug resistance or purine metabolism has been described. Further, SDD-like is not either associated with resistance to toxic nucleosides. Its function is related to synthesis and metabolism of amino acids [44].

Thus, we focused our study on the hypothetical protein LmjF.31.2010. The coding region of this gene was cloned for generating the construct pTRP, which was transfected into LmjF. The protocol for overexpression/selection of transfectants conferred moderate level of resistance to TUB (1.91-fold resistance) compared with that conferred by cosTUB2 (3.78-fold resistance). Despite the low level of resistance mediated by pTRP, there are several indications that *trp* is involved in TUB resistance. First, the signals for protein translation were intact. Second, the levels of resistance were significant for cosTUB2 (with a 30 kb insert), cosTUB2-Δ*Apa*I-III (with a 26 kb insert) and pTRP (with a 3 kb insert). Third, genes that conferred resistance by transfection to other drugs, such as terbinafine and itraconazol [21], vinblastina [45], primaquine [46], pentamidine [30], antimony, miltefosine and amphotericin [41] presented a moderate resistance profile as observed for *trp* gene. As an example, the squalene synthetase gene (*sqs1*) has been identified in a cosmid that conferred resistance to terbinafine (1.47-fold resistance) in *L*. *major* [21].

The analysis of *trp* transcripts revealed that the RNA level was increased in cosTUB2 compared with LmjF. In contrast, we observed similar RNA expression levels when comparing pTRP transcripts. Interesting, the increased resistance level conferred by cosTUB2 (3.78-fold resistance) versus pTRP (1.91-fold resistance) was not correlated with the relative *trp* mRNA abundance. These results indicate that *trp* expression could be subjected to some negative regulation. The increased transcription of *trp* in cosTUB2 transfectants compared with pTRP transfectants can be explained by the action of *cis* and *trans* elements as regulatory factors of mRNA generated by cosTUB2 and pTRP. Moreover, cosmids may contain regulatory sequences that are missing in the plasmid. Indeed, the length of the cosmid DNA can provide a chromatin-like structure that allows to enhance the transcription and consequently promote an increase in protein translation [47].

Western blot analysis revealed that the protein levels differed between LmjF and the transfected lines (cosTUB2 and pTRP), with an increase of TRP expression in the transfected lines. The sub-cellular immunolocalization of TRP in the ER suggests that it is associated with the secretory pathway [48]. Proteins located in the ER may be related to the stress response, the downregulation of translation, spliced leader silencing and protein misfolding [49].

In addition, GFP-tagged TOR has been previously demonstrated to be present at multiple locations in *Leishmania* (i.e., mitochondria and Golgi/trans Golgi regions) except the nucleus [24]. TOR appears to act at the protein level and affect the activities and/or concentration of a class of transporters and other proteins [24]. TUB is transported by adenosine permease, but this purine analog, which is toxic to the parasite, must first enter the cell. According to Detke et al. (2007), parasites become resistant to this toxic purine nucleoside due to the functional loss of the appropriate transporter by mutation or amplification of the *tor* gene, which leads to a decrease of TUB entry into the cell [24].

Nucleoside transport in *Leishmania* is mediate by transporters located in the plasma membrane of the parasite. There are five different members that have different and selective substrate specificities [14]. The regulation of these transporters occurs via salvage pathway because *Leishmania* does not synthesize purine *de novo* [10]. The nucleoside transporters of *L*. *donovani*, LdNT1.1 and LdNT1.2, were previously described. Both transporters mediate adenosine and pyrimidine uptake [15, 50], they are members of the equilibrative nucleoside transporter (ENT) family [51, 52] and exhibit approximately 30% amino acid identity with mammalian ENTs [10]. This low identity is due to differences in the nucleoside transporter members between *Leishmania* and the mammalian host. It has been previously demonstrated that the use of TUB in association with the nucleoside transport inhibitors can result in highly selective toxicity against the parasite, thereby protecting the host against TUB toxicity [20]. LmaNT3 transporter from *L*. *major* was reported with a homology of LdNT1.1 with 33% amino acid sequence identity [53]. It mediates the hypoxanthine, xanthine, adenine and guanine uptake. Interestingly, its functions are optimal at neutral pH in the promastigote form [54]. In contrast, LmaNT4 has very low transport activity at neutral pH, but it is functional in the acid pH that is found within acidified phagolysomal vesicles of host macrophages during the intracellular amastigote stage of the parasite [54].

It was also demonstrated that *Leishmania* overexpressing adenosine permease exhibits an increased sensitivity to TUB [24]. In contrast, resistance to TUB occurs due to functional loss of adenosine and guanosine permease through mutation or amplification of the *tor* gene [22–24]. The reduction in adenosine permease is due to reduction in the amount of transporter *per se* and to the re-routing of the normal trafficking of this transporter from the plasma membrane to the multi-vesicular tubule lysosome [24].

Even in relation to the nucleoside transporters, we hypothesize that the cross-resistance to pentamidine exhibited by cosTUB2 and pTRP transfectants is TRP-dependent. It has been reported that several of the 12 ENTs family identified in *T*. *brucei* with involvement in the salvage pathway can also transport pentamidine [54]. Pentamidine is a second-line drug used as an alternative to the leishmaniasis treatment with pentavalent antimony. The drug enters into the *Leishmania* promastigote or amastigote via a high affinity pentamidine transporter [55]. The mitochondrion is an important target and the drug is involved in the binding and disintegration of kinetoplast DNA [56–58]. Integration of the pathways involved in TUB and pentamidine resistance can be reinforced by the immunolocalization of TRP close to the perinuclear network, although additional studies are required to elucidate this relationship. In contrast, no cross-resistance was observed for allopurinol. Allopurinol is known to inhibit enzymes of the purine salvage pathway in *Leishmania* [59]. The mechanism of action of allopurinol is involved in the conversion to ribonucleoside triphosphate analogs and incorporation into RNA, thereby disrupting macromolecular biosynthesis [60]. Interestingly, TRP overexpression led to a 2-fold increase in allopurinol susceptibility [21].

Three other hypothetical proteins (LmjF.31.2040, LmjF.31.2050 and LmjF.31.2060), non-coding-RNA (LmjF.31.ncRNA) and ubiquitin-fusion protein (LmjF.31.2030) were identified in cosTUB2. Although the term ncRNA is commonly used for RNA that does not encode a protein, it does not mean that the RNA has no genetic information and function. It has been described studies of the involvement of ncRNAs in RNA splicing, editing, translation and turnover [61]. In contrast, ubiquitin is a conserved protein, with a difference of only 3 amino acids between *Saccharomyces cerevisiae* and humans [62]. Protein ubiquitylation is a recognized signal for protein degradation that can control post-translational modifications [55]. It is also known that internalization and retargeting of membrane proteins is frequently initiated by ubiquitination [63, 64]. Although LmjF.31.ncRNA and LmjF.31.2030 were represented in cosTUB2-Δ*Apa*I-I and cosTUB2-Δ*Apa*I-IV, respectively, no TUB resistance *per se* was observed.

We also verified that TRP contains only one hypothetical transmembrane domain and no putative conserved domain. The identification of a single hypothetical transmembrane domain confirms our co-localization, indicating that it is not a membrane transporter, in contrast with the ENTs described in *Leishmania* that contain 11 hypothetical transmembrane domains [52, 65]. The location of the transmembrane domain of TRP in the C-terminal region suggests that this protein can be anchored. Comparative analysis based in TriTrypDB demonstrated that *trp* is specific in the genus *Leishmania*, with no ortholog identified in *T*. *brucei* or *T*. *cruzi*. *In silico* analysis revealed that *trp* gene is located in the same genomic region of chromosome 31 of *L*. *infantum* (LinJ.31.2050), *L*. *tarantolae* (LtaP.31.2440) and *L*. *braziliensis* (LbrM.31.2270).

According to TriTrypDB, *trp* gene is constitutively expressed, however our findings demonstrated that *trp* is more expressed in logarithmic phase. This result can emphasize the likely relation with purine pathway and the potential role of this protein during the replication of the parasite. All these results indicate the importance of characterizing a hypothetical protein not only by functional genomics, but also according to its general biological features, allowing the acquisition of new knowledge about signaling pathways, metabolism, stress response, drug resistance and in the identification of new therapeutic targets. Purine transport can be considered a potential target, since the mechanism of action is different in *Leishmania* and its host. Aoki et al. (2009) demonstrated that the association with a specific inhibitor of the nucleoside transport for mammalian cells, NBMPR, protects infected mammalian host from the toxic effects of TUB. NBMPR inhibits only the mammalian nucleoside transport, thus protecting the host and not the parasite from the TUB toxicity, similarly as proposed in *Schistosoma* model [16, 17].

In conclusion, the TRP, initially annotated as a hypothetical protein was described in this work as involved with TUB resistance.

## Supporting Information

S1 Fig Restriction map of cosTUB1 and functional analysis. Linear representation of cosTUB1 restriction map insert and the four deletions generated by partial digestion with *Kpn*I (K). The restriction sites of *Eco*RI (EI) are also indicated in the figure. TUB resistance is indicated by (+) sign. The white arrows indicate the coding region of the peptidase m20/m25/m40 family-like protein (LmjF.31.1890), the hypothetical proteins (LmjF.31.1910 and LmjF.31.1920), and the dihydrouridine synthase (DUS) (LmjF.31.1930). The black arrow indicates the transcription-factor-like protein (nupM1) (LmjF.31.1940), also known as TOR. The shaded boxes represent the cLHYG vector.
(TIF)

S2 Fig Alignment of the transcription-factor-like protein (LmjF.31.1940) from *L*. *major* with their orthologs of *Leishmania* spp. Multiple alignment of amino acid sequence of the transcription-factor-like protein of *L*. *major* Friedlin (LmjF), *L*. *(L*.*) tarentolae* Parrot-TarII (LtaP), *L*. *(V*.*) braziliensis* MHOM/BR/75/M2904 (LbrM), *L*. *(L*.*) infantum* JPCM5 (LinJ), *L*. *(L*.*) mexicana* MHOM/GT/2001/U1103 (LmxM), *L*. *(L*.*) donovani* BPK282A1 (LdBPK). The identical amino acids are highlighted in gray and the transmembrane domain is boxed.
(TIF)

S3 Fig Evaluation of TRP in mRNA transcripts expression and protein expression levels during time-course of *L*. *major* growth curve. (A) Symmetrical sigmoidal nonlinear regression curve of LmjF during time-course of growth. The values are the mean ± SEM of three independent biological preparations. (B) TRP mRNA expression level of promastigotes LmjF. Data were based on the quantification of target and normalized by GAPDH data. (*) p < 0.005, (**) p < 0.002, (***) p < 0.001, compared the day 3 to that on days 5, 7 and 9, respectively. The values are the mean ± SEM of three independent biological preparations. (C) Western blot analysis of total extract of promastigotes during time-course of LmjF. The extracts were lysed and then proteins were separated by SDS-PAGE, transferred to nitrocellulose membrane and immunoblotted with anti-TRP polyclonal antibody. The anti-α-tubulin antibody was used as a control. The images were scanned using an Odyssey CLx imaging system (Li-COR). (D) The bands were quantified using Image Studio2.1 Software (Li-COR), and the results for TRP were normalized against α-tubulin for blotting comparisons. Statistical analysis was performed using Mann-Whitney U test. (*) p < 0.05, (**) p < 0.001, comparing the day 3 with days 5, 7 and 9.
(TIF)

S4 Fig Alignment of TRP (LmjF.31.2010) from *L*. *major* with their orthologs of *Leishmania* spp. Multiple alignment of amino acid sequence of the TRP of *L*. *major* Friedlin (LmjF), *L*. *(L*.*) tarentolae* Parrot-TarII (LtaP), *L*. *(V*.*) braziliensis* MHOM/BR/75/M2904 (LbrM), *L*. *(L*.*) infantum* JPCM5 (LinJ), *L*. *(L*.*) mexicana* MHOM/GT/2001/U1103 (LmxM) and *L*. *(L*.*) donovani* BPK282A1 (LdBPK). The identical amino acids are highlighted in gray and the transmembrane domain is boxed.
(TIF)

S1 Table TUB resistance profiles of cosTUB1 and its respective deletions compared with LmjF parasites. (DOCX)
