# Supplementary figures and images for: Characterization of a Novel Endoplasmic Reticulum Protein Involved in Tubercidin Resistance in Leishmania major
Source: PLoS Negl Trop Dis. 2016 Sep 8;10(9):e0004972. doi: 10.1371/journal.pntd.0004972 (PMC5015992; doi:10.1371/journal.pntd.0004972)

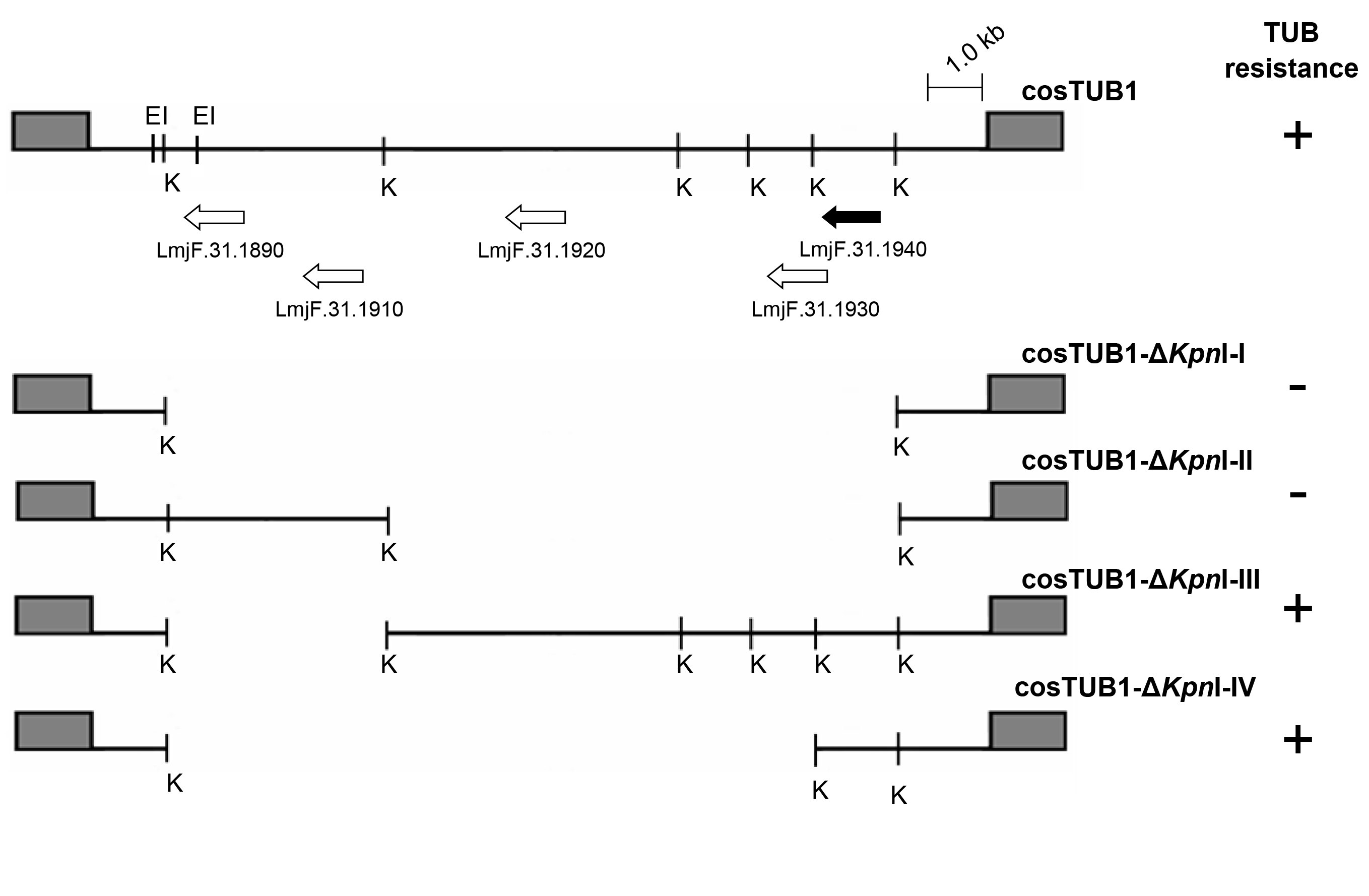

Supplement: S1 Fig — Linear representation of cosTUB1 restriction map insert and the four deletions generated by partial digestion with KpnI (K). The restriction sites of EcoRI (EI) are also indicated in the figure. TUB resistance is indicated by (+) sign. The white arrows indicate the coding region of the peptidase m20/m25/m40 family-like protein (LmjF.31.1890), the hypothetical proteins (LmjF.31.1910 and LmjF.31.1920), and the dihydrouridine synthase (DUS) (LmjF.31.1930). The black arrow indicates the transcription-factor-like protein (nupM1) (LmjF.31.1940), also known as TOR. The shaded boxes represent the cLHYG vector. (TIF) [file pntd.0004972.s001.tif]

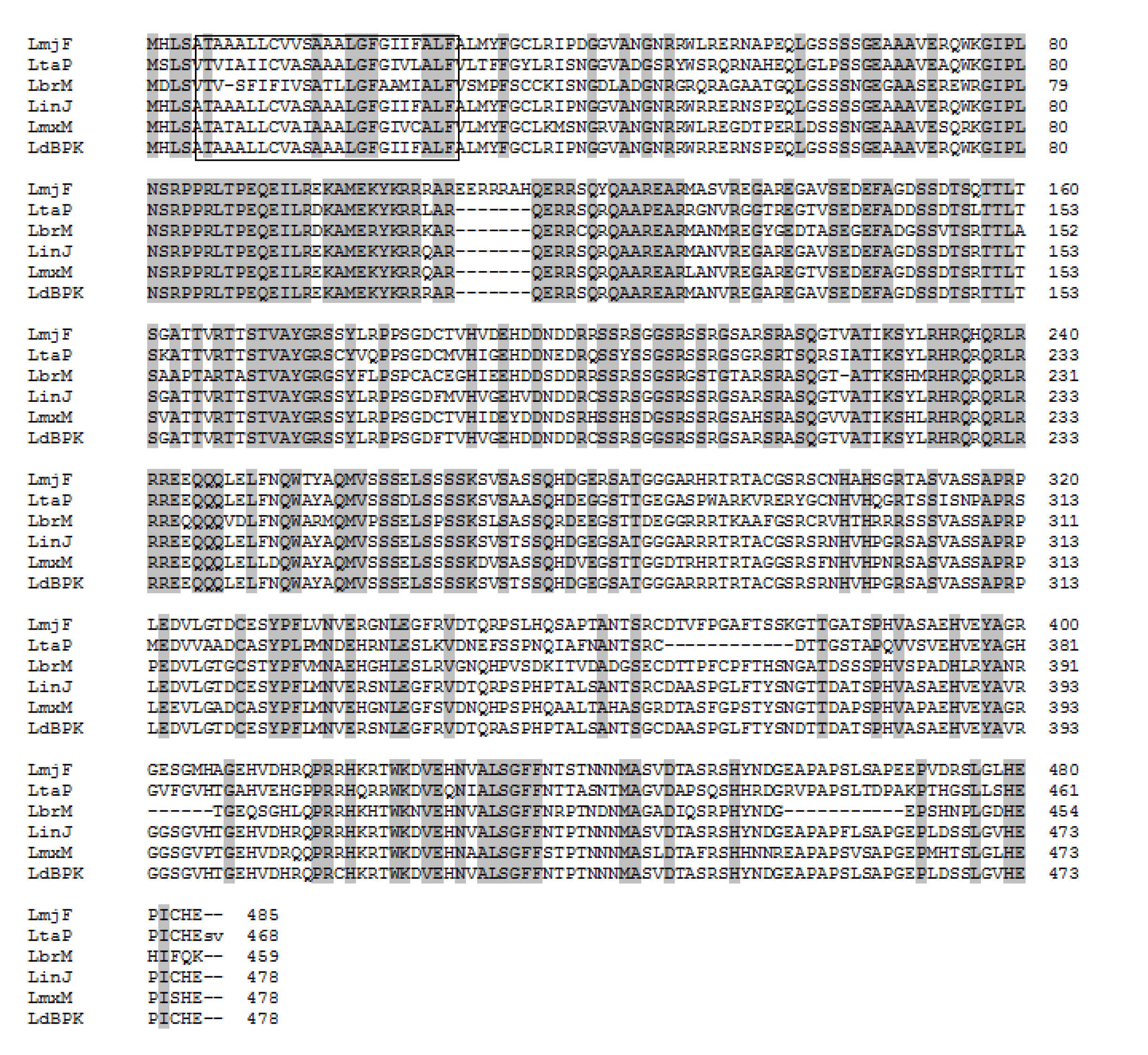

Supplement: S2 Fig — Multiple alignment of amino acid sequence of the transcription-factor-like protein of L. major Friedlin (LmjF), L. (L.) tarentolae Parrot-TarII (LtaP), L. (V.) braziliensis MHOM/BR/75/M2904 (LbrM), L. (L.) infantum JPCM5 (LinJ), L. (L.) mexicana MHOM/GT/2001/U1103 (LmxM), L. (L.) donovani BPK282A1 (LdBPK). The identical amino acids are highlighted in gray and the transmembrane domain is boxed. (TIF) [file pntd.0004972.s002.tif]

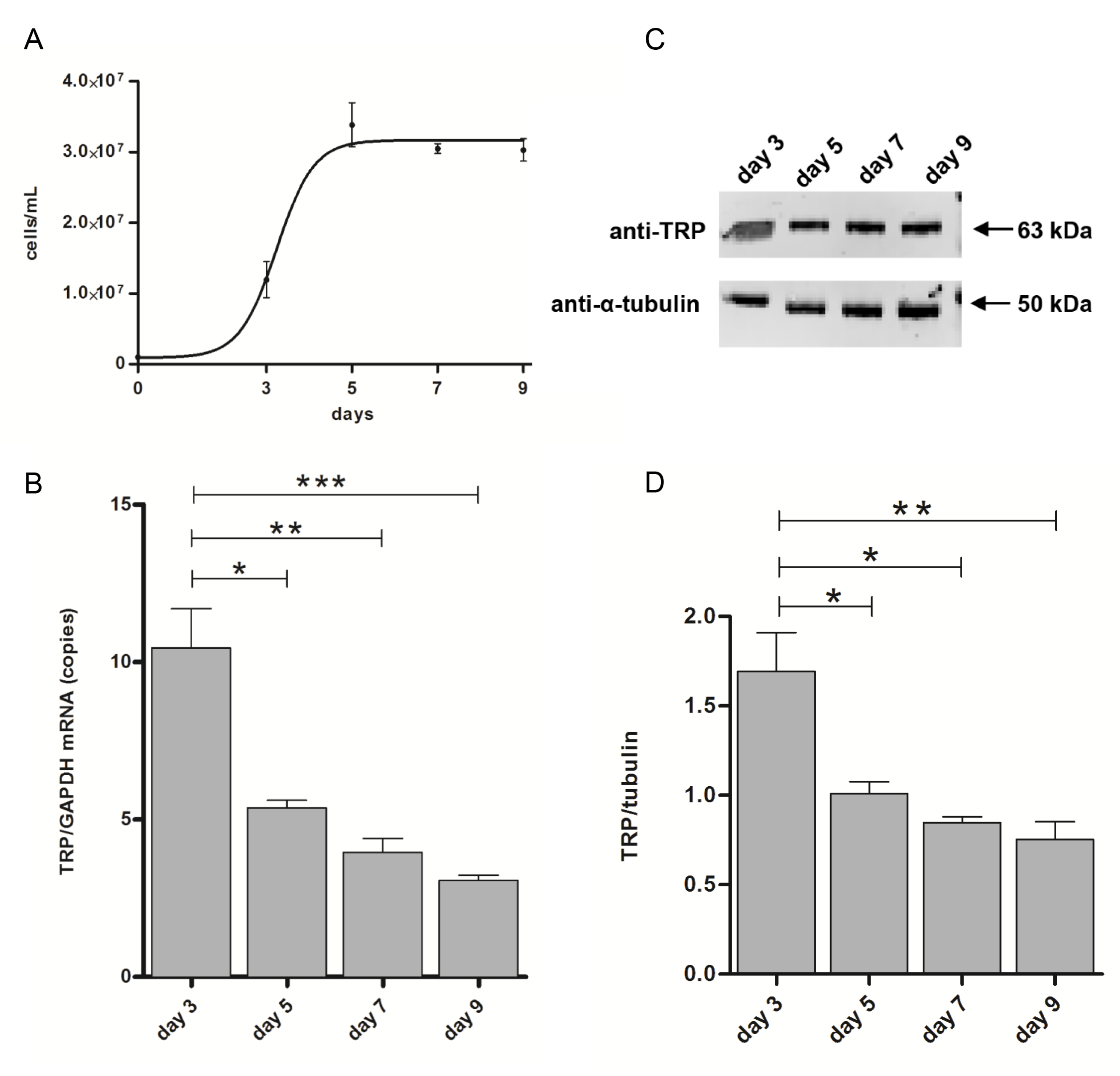

Supplement: S3 Fig — (A) Symmetrical sigmoidal nonlinear regression curve of LmjF during time-course of growth. The values are the mean ± SEM of three independent biological preparations. (B) TRP mRNA expression level of promastigotes LmjF. Data were based on the quantification of target and normalized by GAPDH data. (*) p < 0.005, (**) p < 0.002, (***) p < 0.001, compared the day 3 to that on days 5, 7 and 9, respectively. The values are the mean ± SEM of three independent biological preparations. (C) Western blot analysis of total extract of promastigotes during time-course of LmjF. The extracts were lysed and then proteins were separated by SDS-PAGE, transferred to nitrocellulose membrane and immunoblotted with anti-TRP polyclonal antibody. The anti-α-tubulin antibody was used as a control. The images were scanned using an Odyssey CLx imaging system (Li-COR). (D) The bands were quantified using Image Studio2.1 Software (Li-COR), and the results for TRP were normalized against α-tubulin for blotting comparisons. Statistical analysis was performed using Mann-Whitney U test. (*) p < 0.05, (**) p < 0.001, comparing the day 3 with days 5, 7 and 9. (TIF) [file pntd.0004972.s003.tif]

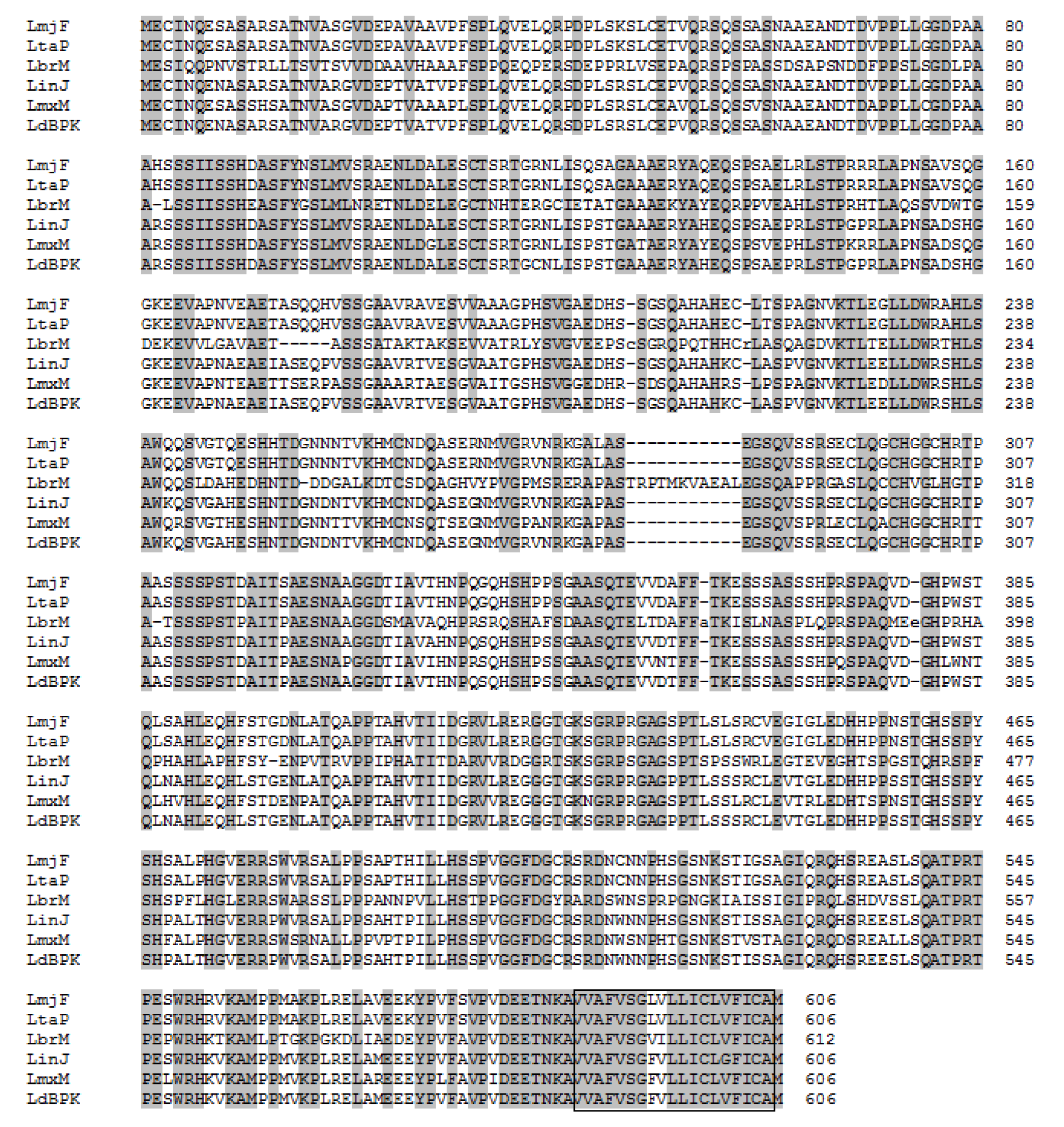

Supplement: S4 Fig — Multiple alignment of amino acid sequence of the TRP of L. major Friedlin (LmjF), L. (L.) tarentolae Parrot-TarII (LtaP), L. (V.) braziliensis MHOM/BR/75/M2904 (LbrM), L. (L.) infantum JPCM5 (LinJ), L. (L.) mexicana MHOM/GT/2001/U1103 (LmxM) and L. (L.) donovani BPK282A1 (LdBPK). The identical amino acids are highlighted in gray and the transmembrane domain is boxed. (TIF) [file pntd.0004972.s004.tif]
