# Supplementary material for: Characterization of a Novel Endoplasmic Reticulum Protein Involved in Tubercidin Resistance in Leishmania major
Source: PLoS Negl Trop Dis. 2016 Sep 8;10(9):e0004972. doi: 10.1371/journal.pntd.0004972 (PMC5015992; doi:10.1371/journal.pntd.0004972)
Supplement: S1 Table — (DOCX) [file pntd.0004972.s005.docx]

**S1 Table. TUB resistance profiles of cosTUB1 and its respective deletions compared with LmjF parasites**

| Cell line | IC_50_ ^(a)^  (µM) | fold ^(b)^  resistance | p ^(c)^ | TUB  resistance |
| --- | --- | --- | --- | --- |
| LmjF | 0.23 ± 0.09 | - | - | no |
| cLHYG | 0.23 ± 0.04 | 1.00 | ns | no |
| cosTUB1 | 0.45 ± 0.05 | 1.95 | 6x10^-4^ | yes |
| cosTUB1-Δ*Kpn*I-I | 0.27 ± 0.03 | 1.17 | ns | no |
| cosTUB1-Δ*Kpn*I-II | 0.30 ± 0.02 | 1.34 | ns | no |
| cosTUB1-Δ*Kpn*I-III | 0.47 ± 0.02 | 2.04 | 6x10^-4^ | yes |
| cosTUB1-Δ*Kpn*I-IV | 0.65 ± 0.07 | 2.82 | 2x10^-6^ | yes |

^(a)^ Mean ± standard deviation of the IC_50_ values of at least three independent experiments for each indicated cell line.

^(b)^ Fold-resistance is the ratio of the IC_50_ value for the transfected lines and for LmjF WT.

^(c)^ *p* values versus the LmjF according to the non-parametric Kruskal-Wallis test.

(ns) – not significant.
